# A realistic assessment of methods for extracting gene/protein interactions from free text

**DOI:** 10.1186/1471-2105-10-233

**Published:** 2009-07-28

**Authors:** Renata Kabiljo, Andrew B Clegg, Adrian J Shepherd

**Affiliations:** 1 School of Crystallography and Institute of Structural and Molecular Biology, Birkbeck College, University of London, Malet Street, London WC1E 7HX UK; 2 Research Department of Structural and Molecular Biology and Institute of Structural and Molecular Biology, University College London, Gower St, London, WC1E 6BT UK

## Abstract

**Background:**

The automated extraction of gene and/or protein interactions from the literature is one of the most important targets of biomedical text mining research. In this paper we present a realistic evaluation of gene/protein interaction mining relevant to potential non-specialist users. Hence we have specifically avoided methods that are complex to install or require reimplementation, and we coupled our chosen extraction methods with a state-of-the-art biomedical named entity tagger.

**Results:**

Our results show: that performance across different evaluation corpora is extremely variable; that the use of tagged (as opposed to gold standard) gene and protein names has a significant impact on performance, with a drop in F-score of over 20 percentage points being commonplace; and that a simple keyword-based benchmark algorithm when coupled with a named entity tagger outperforms two of the tools most widely used to extract gene/protein interactions.

**Conclusion:**

In terms of availability, ease of use and performance, the potential non-specialist user community interested in automatically extracting gene and/or protein interactions from free text is poorly served by current tools and systems. The public release of extraction tools that are easy to install and use, and that achieve state-of-art levels of performance should be treated as a high priority by the biomedical text mining community.

## Background

The automated extraction of gene and/or protein interactions (GPIs) from the literature is one of the most important targets of biomedical text mining research. From a biological standpoint, there are several distinct sub-types of GPI, including the direct physical interactions between proteins, protein-DNA interactions (notably the binding of transcription factors to DNA sequences), and the encoding of a given protein by a specific gene. However, in the biomedical text mining community it is common practice to treat all these interactions as belonging to a single task – here termed the GPI extraction task.

By providing a publicly-available mechanism for converting multiple GPI corpora to a common format, Pyysalo and co-workers have made it comparatively easy to undertake rich analyses of the performance of GPI extraction tools [1] comparable to those already undertaken for named-entity recognition tools [2,3].

Included within the paper by Pyysalo and co-workers is an assessment of the performance of the RelEx GPI tool [4] and a simple co-occurrence based method. Although the analysis is both interesting and instructive, it is primarily intended to shed light on the differences between GPI corpora and does not, we believe, provide a realistic assessment of GPI tool performance for the following reasons:

• The analysis uses the gold standard named entities (mainly the names of genes and proteins) as annotated within the corpora, and hence does not take into account the effects of named entity recognition (NER) errors.

• The authors re-implemented RelEx (as a public implementation was not available), whereas a typical non-specialist user will prefer off-the-shelf tools that are relatively easy to install and use.

The use of gold standard annotations in the evaluation of GPI methods is commonplace. This includes shared tasks such as the LLL challenge, for which the exact location of the target entities within the text was provided in advance [5]. For the GPI pairs sub-task at BioCreAtIvE II, a list of relevant gene mention symbols and synonyms was provided in advance [6].

However, this evaluation protocol is potentially highly misleading, as the performance scores it awards to GPI algorithms are rather inflated. Our previous analyses of gene/protein tagger performance have shown that exact matching to the boundaries of the manually-annotated entities in a range of corpora is around 50–60% with the best performing taggers [2,3]. An evaluation of the impact of tagger errors on the performance of GPI methods is one of the main goals of this paper.

Here we present an evaluation of several GPI methods coupled with a state-of-the-art entity tagger on five GPI corpora. In addition to assessing the performance of each method, we consider how much effort is involved in setting it up and using it, as we believe this is a key issue for most non-specialist users. Taken as a whole, we believe our analysis represents the first realistic evaluation of GPI extraction methods, and sheds light on their performance in a way that is directly relevant to both users and developers.

## Methods

### GPI corpora

The five GPI corpora used in this evaluation were: the AIMed corpus [7], the BioInfer corpus [8], the HPRD50 corpus [4], the IEPA corpus [9], and the LLL training corpus, a GPI corpus produced for the LLL challenge [5]. Here we provide a short summary of the five corpora. For a more detailed comparison, see [1].

The AIMed corpus contains 225 abstracts manually annotated for interactions between human genes and proteins. Most of the abstracts contain interactions, but a significant percentage (around 10%) do not, and were deliberately added to provide negative examples. The HPRD50 corpus contains 50 abstracts in which human gene and protein names were automatically identified using the ProMiner protein and gene name tagger [10]. The IEPA (Interaction Extraction Performance Assessment) corpus contains 303 abstracts from PubMed, each containing a specific pair of co-occurring chemicals obtained using 10 queries chosen to represent diverse biological research topics. The LLL corpus was created as the shared dataset for the Learning Language in Logic 2005 (LLL05) challenge and contains 77 sentences. The domain of LLL is gene interactions of Bacillus subtilis. The BioInfer corpus consists of 1100 sentences from PubMed abstracts that contain at least one pair of interacting genes or proteins. All protein, gene and RNA entities were manually annotated, together with all interactions between these entities, including static relations. Each interaction is mapped to the Bioinfer relationship ontology, defined especially for this purpose. BioInfer permits the annotation of relationships with a complex structure, such as relationships between relationships, or relationships of more than two entities.

These corpora differ significantly in their working definitions of the concept "gene/protein interaction". For example, in the IEPA corpus an interaction is a "direct or indirect influence of one on the quantity or activity of the other" [9], whereas BioInfer additionally contains so-called "static" entity relationships, such as family membership. Nevertheless, an analysis by Pyysalo and co-workers has shown that "a clear majority of all interactions [in these corpora]... correspond to events occurring as part of biochemical processes in living cells", as opposed to static relationships [1]. A more recent paper by Pyysalo and a different set of co-workers advocates addressing the extraction of static relationships as a distinct subtask [11], but this is not tackled by existing publicly-available tools.

For our analysis we converted all five corpora to a unified format using the conversion software provided by Pyysalo and co-workers [1]. To simplify our analysis, all 68 sentences in the BioInfer corpus that contain at least one discontinuous entity were discarded. For example, in the phrase 'myosin heavy chain and light chains', the annotated entities are 'myosin heavy chain' and 'myosin light chains', although the latter does not appear as a continuous string in surface text.

### Gene/protein taggers

In two earlier papers we concluded that the version of ABNER [12] trained on the BioCreAtIvE corpus [13] was the best performing tagger on a range of biomedical corpora [2] and on a new corpus – ImmunoTome – consisting of ten full-text immunological articles [3].

However, since the publication of those papers, we have evaluated BANNER, a new biomedical named-entity recognition system implemented using conditional random fields [14]. BANNER exploits a range of orthographic, morphological and shallow syntax features, such as part-of-speech tags, capitalisation, letter/digit combinations, prefixes, suffixes and Greek letters. As with the best-performing version of ABNER, BANNER was trained on the BioCreAtIvE corpus.

As shown in tables 1 and 2, BANNER consistently outperforms ABNER on the same corpora used in our earlier evaluations (Yapex [15], GENIA [16], ProSpecTome [2] and ImmunoTome [3]), and has therefore been used for the analysis of GPI methods we present here.

**Table 1:** The F-scores produced by ABNER and BANNER when applied to four corpora using sloppy matching criteria.

	Y	J	P	I
ABNER(B)	80.4	76.0	85.3	78.3

BANNER	85.0	77.5	89.7	83.4

Abbreviations are as follows: Y = Yapex; J = JNLPBA evaluation corpus; P = ProSpecTome; I = ImmunoTome; B = BioCreAtivE.

**Table 2:** The F-scores produced by ABNER and BANNER when applied to four corpora using strict matching criteria.

	Y	J	P	I
ABNER(B)	54.2	60.8	62.0	54.0

BANNER	62.0	61.0	68.7	53.9

Abbreviations are as follows: Y = Yapex; J = JNLPBA evaluation corpus; P = ProSpecTome; I = ImmunoTome; B = BioCreAtivE.

### GPI extraction methods

A number of different GPI extraction methods have been published in the literature (for recent discussions of published methods see [17] and [18]), with some 26 teams submitting runs for at least one of the GPI annotation extraction tasks at BioCreAtIvE II [6].

However, our purpose here was to undertake an evaluation of the state-of-the-art in GPI extraction relevant to potential non-specialist users. In contrast to entity taggers, a number of which are easy to install locally or can be accessed directly via the Web, none of the GPI extraction methods are trivial to install and use. This is partly a consequence of the complex, modular nature of a typical state-of-the-art GPI method that combines third-party components (a part-of-speech tagger and one or more parsers) with a machine learning or rule-based algorithm for identifying possible relationships within a given parse. As noted in [17], the vast majority of such GPI methods are currently not publicly available.

Here we focus on four GPI methods: AkanePPI, Whatizit, OpenDMAP, and a simple benchmark approach that we developed ourselves using Perl regular expressions. One system we have not evaluated, even though it is designed primarily for non-specialists and is easy to use, is iHOP [19]. iHOP is a dictionary-based system that uses genes and proteins as hyperlinks between sentences and abstracts in order to navigate information in PubMed. When it comes to GPI, for every gene detected in a query, there is a link that leads to sentences (and subsequently abstracts) which describe interactions of that gene with other genes. However, iHOP does not accept text submitted by the user, making it unsuitable for the analyses we undertook for this paper.

AkanePPI [20] is a state-of-the-art GPI method for which the C++ source code is publicly available. AkanePPI combines the version of the deep syntactic parser Enju that has been retrained on the GENIA corpus [21] with a shallow dependency parser [22]. A support vector machine with tree kernels [23] is used to extract rules for identifying pairs of interacting genes/proteins from a training corpus. Here we used two versions of AkanePPI, the original, distributed version (AkanePPI(A)) trained on the AIMed corpus, and a second version ((AkanePPI(B)) we retrained ourselves on the BioInfer corpus. The authors report an F-score of 52% for GPI extraction from unseen abstracts [20].

OpenDMAP [24] is a general-purpose parsing and information extraction platform that provides an Open Source Java API. It was adapted to perform GPI extraction for the Protein Interaction Pairs subtask at BioCreAtIvE II [6], where it outperformed other participating systems, achieving precision of 39% and recall of 31% when scores were averaged over articles [25]. OpenDMAP uses a rule-based approach. For BioCreAtIvE II, patterns were devised manually from the BioCreAtIvE, PICorpus [26] and Prodisen [27] corpora in consultation with biologists. These patterns have been made available for download together with the main distribution and have been used here.

Whatizit [28] is a modular text processing system available through the EBI website. Of the wide range of text mining services on offer, here we focused exclusively on the protein interaction pipeline. (The core pipeline is available separately from Whatizit in the form of the Protein Corral web application [29]. However, Protein Corral is designed to perform Medline searches and does not accept text submitted by the user.) The pipeline begins by mapping gene/protein names to UniProt identifiers using dictionary look-up. It then attempts to identify relationships between any successfully mapped names using three approaches of decreasing precision, but increasing coverage: natural language processing (Ppi); the co-occurrence of two gene/protein names with an interaction verb (Co3); and the co-occurrence of two names without an interaction verb (Co). The abbreviations here are the ones used on the Protein Corral website.

Finally, we developed our own simple baseline method using Perl regular expressions. Every time two gene/protein names occur together within a sentence and have an interaction keyword between them they are predicted to be an interacting pair of genes/proteins. A minority of the interaction words were inherited from two earlier projects – GIFT [30] and GraphSpider [31]. The former derived its verb list from FlyBase [32] and the latter from the LLL training corpus. The remaining verbs were obtained semi-automatically using the *clueType *event attribute in the GENIA event corpus [33]. Our list of interaction keywords and Perl script are available as supplementary material [see Additional file 1 and Additional file 2 respectively].

In addition, we compare the performance of our baseline method with the simpler co-occurrence baseline previously used by Pyysalo and co-workers, which predicts an interaction between every pair of genes/proteins co-occurring in a sentence irrespective of whether an interaction verb is present [1]. To easily distinguish between these two baseline methods within this paper, we call our keyword baseline method Baseline(K) and the simple co-occurrence baseline method of Pyysalo *et al*. Baseline(C).

### Evaluating performance

With the unified corpus format used here, all interactions are both undirected and binary. Hence, when the gold standard named entities are used, scoring is straightforward – either a given interaction has been predicted, or it has not. However, when a tagger is used to identify putative gene/protein entities, there is more than one way to score predicted interactions.

Elsewhere we have argued that "sloppy" matching criteria (where the tagger scores a "hit" provided part of the name is matched) provide a fairer evaluation of tagger performance than "strict" matching criteria (where the tagger is required to match a given name exactly to score a "hit"), as the latter is more sensitive to the essentially arbitrary choices made when drawing up annotation guidelines for the evaluation corpora – for example, whether the word "mouse" is part of the protein name in the phrase "mouse oxytocin" [3].

In the context of GPI extraction, the use of "sloppy" matching criteria is somewhat more complex, as the following exemplar sentence from the LLL corpus illustrates:

Three new *sigmaB*-dependent genes (*ydaE, ydaG *and *yfkM*) encoding proteins with still unknown functions were also described.

In the LLL corpus, three interactions are annotated within this sentence – *sigmaB *with *ydaE*, *ydaG *and *yfkM *respectively. However, we found that BANNER tagged the phrase "sigmaB-dependent genes" as a single entity, an entity that has no interactions with *ydaE*, *ydaG *and *yfkM *(they are simply instances of that entity). In this example, it would be misleading to penalize the GPI extraction method for failing to identify the three interactions in the LLL corpus if it was using the entities identified by BANNER.

The question arises, therefore, whether the use of sloppy matching criteria for gene/protein names is, on balance, more informative than strict matching criteria in the context of GPI extraction. To investigate this issue, we manually inspected those interactions extracted by BANNER and AkanePPI(A) from the two smallest corpora that count as misses using strict criteria but hits using sloppy criteria. Out of 30 such interactions from the LLL corpus, we judged 24 to be valid interactions and six to be erroneous. Of these six, two had erroneous names (e.g. "sigB mutant probably via sigma(H)"), whereas in the remaining four the names were valid, but the interactions invalid. For example, for the sentence from LLL given in the preceding paragraph, AkanePPI wrongly predicted that the tagged entity "sigmaB-dependent genes" interacts with "ydaG" and "yfkM". Out of 20 such interactions from the HRPD50 corpus, we judged 16 to be valid interactions and four to be erroneous. Of these four, all are attributable to invalid names being tagged by BANNER (for example "1C" instead of "protein tyrosine phosphatase 1C", "GC" and "GAP" instead of "GC-GAP").

To quantify this effect, we calculated the corrected F-score for the two corpora by taking account of the false positives uncovered during our manual analysis. For LLL, the corrected F-score was 4 percentage points lower than the sloppy criteria F-score and 20 percentage points higher than the strict criteria F-score. For HPRD50, the corrected F-score was 2 percentage points lower than the sloppy criteria F-score and 27 percentage points higher than the strict criteria F-score. Consequently, we conclude that sloppy matching criteria are significantly more informative than strict matching criteria, and it is the results for sloppy criteria that we report in this paper.

## Results and discussion

### Ease of usage

We begin by considering how easy each of the major GPI tools discussed in this paper – AkanePPI, Whatizit and OpenDMAP – is to install and use.

Setting up the AkanePPI system involves downloading and compiling the C++ source code. On running the make script provided with the distribution, several components are retrieved from their respective websites, notably the Enju parser [34], TinyXML [35], SVMlight with Tree Kernels [36], and Standoff Manager [37]. We found AkanePPI easy to build and run on the pre-parsed example supplied, but installing the Enju parser was non-trivial owing to problems encountered with environment variables and library dependencies. Enju only claims Linux compatibility, consequently we were unable to build it on a Mac, despite AkanePPI advertising Mac compatibility.

AkanePPI is supplied with a configuration file tailored to the AIMed corpus. Although this could be modified to improve the performance of AkanePPI on different data, most of the settings require linguistic expertise to understand how this might be achieved. For this paper we have used the AIMed configuration file with only trivial modifications (reflecting the names and locations of files on our local system), as we are primarily interested in evaluating tools from the perspective of the general user.

Via its web interface, the Whatizit protein interaction pipeline is easy to use, but the output does not specify what interactions are present (it merely tags entity names and interaction verbs), nor does it specify interaction confidence levels. However, both of these output features are available when the pipeline is used as a webservice or servlet. The example Java client provided is straightforward to adapt given moderate proficiency in writing Java. Output from the pipeline is in XML (we encountered some problems with mismatching XML tags).

Although arguably sensible for some applications, the requirement that gene/protein names are mapped to UniProt identifiers makes the Whatizit protein interaction pipeline different to the other systems evaluated here. It is not possible to use pre-tagged entities with the pipeline, be they the gold-standard corpus entities used in our evaluation, or those generated by a state-of-the-art tagger such as BANNER. Moreover, in the case where the same protein name occurs more than once in a single sentence, the pipeline does not specify which of the names it has identified as providing evidence that an interaction is occurring. (Our analysis shows that such sentences are surprisingly common, accounting for between 14% and 24% of sentences in the five GPI corpora used here.) This may be of little practical significance, but it does necessitate the use of weaker scoring criteria than are generally applied to the evaluation of GPI extraction methods. Consequently we excluded Whatizit from most of the comparative evaluations undertaken for this paper, although we do separately assess its performance on our chosen GPI corpora.

It is also worth noting that, as the Whatizit protein interaction pipeline is a remotely-hosted service (unlike the other tools evaluated here), the user is dependent on the reliability of third-party service provision. Our experience was that the service was unavailable for several days during our evaluation period lasting several weeks.

Installing OpenDMAP was reasonably straightforward but not entirely trivial. It is supplied as a tarball containing the Java source, technical documentation, etc., plus a pre-compiled binary. As well as the main distribution, the user must obtain a pair of JAR files from the Protégé [38] distribution, and for GPI extraction, a set of supplementary patterns (originally designed for the BioCreAtIvE PPI sub-task) that are provided separately. To configure OpenDMAP to use these patterns also requires an XML configuration file, but this is not supplied with the patterns, so we wrote our own by reference to one of the examples provided with the main distribution.

Submitting arbitrary text with marked-up entities is not a trivial task either, and we were able to achieve this only with help from the authors, including some sample code custom-written for our requirements. Our experiences are consistent with the fact that OpenDMAP is not designed as a PPI/GPI application for biology or bioinformatics researchers, even with the availability of the BioCreAtIvE project files, but rather as an extensible tool for NLP research and language engineering.

Of the tools evaluated for this paper, the web interface to the Whatizit protein interaction pipeline is by far the easiest to use, but also somewhat restricted; it will not suit all potential users and applications. In the case of all other tools, none proved entirely trivial to download and use. Indeed, in every case we found it necessary to contact the authors in order to get the tool to work properly. Our conclusion is that, with the notable exception of Whatizit, the vast majority of biologists will not be able to install and use these tools – in spite of the fact that biologists are one of the most important groups of potential users for this kind of tool.

### The performance of BANNER

Before moving on to evaluate the performance of the GPI extraction tools on the GPI corpora, it is useful to consider how well the gene/protein name tagger BANNER performs on these corpora. The performance of BANNER is summarized in Table 3. Here BANNER is shown to perform best on BioInfer, the corpus with by far the highest annotation density [1]. The poor performance on IEPA is attributable to the fact only a subset of the gene and protein names in this corpus are annotated (reducing BANNER's precision), whilst the labelled entities in this corpus include cholesterol, gibberellins, and flavonoids in addition to genes and proteins (reducing BANNER's recall).

**Table 3:** The (P)recision, (R)ecall and (F)-measure scores for BANNER when applied to five GPI corpora.

	A	B	H	I	L
P	80.5	97.5	70.8	60.4	80.0

R	85.4	85.1	83.2	69.6	88.7

F	82.9	90.8	76.5	64.6	84.1

Corpus abbreviations are as follows: A = AIMed; B = BioInfer; H = HPRD50; I = IEPA; L = LLL.

This level of performance is broadly comparable to that for standard gene/protein NER corpora, as reported in table 1.

### The performance of GPI extraction methods with gold-standard entities

The performance of our chosen GPI extraction methods on the five GPI corpora with gold-standard named-entity annotations is summarized in Table 4. In terms of F-score, the key features of these results are as follows:

**Table 4:** The performance (precision, recall and F-score) of six GPI extraction methods when applied to five GPI corpora using gold-standard named entities.

	A	B	H	I	L
**Precision:**					

AkanePPI(A)	(57.0)	29.2	61.5	60.2	69.6

AkanePPI(B)	29.1	(56.8)	52.0	66.2	76.7

RelEx	40	39	76	74	82

Baseline(K)	22.8	24	54	44.8	(53.9)

Baseline(C)	17	13	38	41	50

OpenDMAP	61	62.3	77.3	87.5	100

**Recall:**					

AkanePPI(A)	(74.0)	31.8	44.2	32.5	23.8

AkanePPI(B)	52.9	(85.4)	55.8	51.3	40.2

RelEx	50	45	64	61	72

Baseline(K)	51.5	52.2	66.9	56.4	(72)

Baseline(C)	95	99	100	100	100

OpenDMAP	9.1	5.9	10.4	2.1	2.4

**F-score:**					

AkanePPI(A)	(64.4)	30.5	51.4	42.2	35.4

AkanePPI(B)	37.5	(68.2)	53.8	57.8	52.8

RelEx	44	41	69	67	77

Baseline(K)	31.6	32.9	59.7	49.9	(61.6)

Baseline(C)	29	23	55	58	66

OpenDMAP	15.9	10.8	18.4	4.1	4.8

The figures for RelEx and Baseline(C) are taken from Pyysalo *et al*. (2008). (Note that we use a simplified version of BioInfer compared to the one used in that paper, so the figures for this corpus are not completely comparable.) Figures are given in brackets where a corpus was used to develop a given method. Corpus abbreviations are as follows: A = AIMed; B = BioInfer; H = HPRD50; I = IEPA; L = LLL.

• The best method is the rule-based RelEx, which is not, however, publicly available.

• Although OpenDMAP has the highest precision, it has by far the lowest coverage leading to the worst over all performance on all corpora. Given that its coverage is so low (ranging from 2.1% to 10.4%), we have largely excluded it from our subsequent analyses. Its poor performance is most likely attributable to the BioCreAtivE pattern set being optimized specifically for protein-protein interactions (whereas our chosen evaluation corpora annotate a mixture of gene and protein interactions), rather than being a fundamental characteristic of the underlying approach – there are no patterns in this set based around words like "transcribed", "express" or "induction" which are very common in sentences describing gene regulation events. However, OpenDMAP's BioCreAtivE entry showed highly variable results even on this more constrained topic, where its interaction extraction F-score on its own training data was only 7.8% [25].

• AkanePPI trained on BioInfer performs significantly better than AkanePPI trained on AIMed. In this context it is worth noting that our earlier experience with the tagger ABNER indicates that the choice of training corpus can have a significant impact on the performance of a text-mining tool [2,3].

• Our simple keyword-based baseline approach, Baseline(K), performs surprisingly well. It out-performs the standard version of AkanePPI (i.e. the version trained on AIMed) on four of the five corpora. It also out-performs the simpler co-occurrence baseline approach, Baseline(C), on three of the corpora.

• The performance of all GPI extraction methods except OpenDMAP is broadly correlated across all corpora. Scores are consistently worst on BioInfer and AIMed, and significantly higher on the other three corpora.

However, this is only part of the story, as the GPI extraction methods differ significantly in their relative performance with respect to precision and recall. Broadly speaking, the two baselines are high-recall, low-precision methods, whereas OpenDMAP and AkanePPI(A) are low-recall, high-precision. Only the two methods that are not publicly available, AkanePPI(B) and RelEx are reasonably balanced in their precision/recall performance.

Finally, we wished to estimate the significance of removing the 68 sentences containing discontinuous entities from the BioInfer corpus. To do this we evaluated the performance of AkanePPI(A) on the entire BioInfer copus. The result was that the F-measure dropped by 0.7 percentage points. This suggests that removing these sentences does not have a large impact on the validity of the reported scores.

### Joint error analysis

To understand the nature of the GPI extraction errors and whether they are correlated between different tools, we undertook a joint error analysis for AkanePPI(A), AkanePPI(B) and Baseline(K). (OpenDMAP was excluded from this analysis on the grounds of its exceptionally low coverage.) Given that the two versions of AkanePPI were trained on AIMed and BioInfer respectively, whereas Baseline(K) incorporates keywords from the LLL corpus, we have performed our analysis on the only corpora that represent unseen data for all three methods: HPRD50 and IEPA.

Of the 163 interactions in the HPRD50 corpus, 39 were detected by all three methods and 16 missed by all three. There were 6 false positive interactions common to all three methods. The number of true positive interactions identified by each method alone were 14 (AkanePPI(A)), 33 (AkanePPI(B)) and 40 (Baseline(K)) respectively.

Of the 335 interactions in the IEPA corpus, 52 were detected by all three methods and 43 missed by all three. There were 8 false positive interactions common to all three methods. The number of true positive interactions identified by each method alone were 40 (AkanePPI(A)), 90 (AkanePPI(B)) and 78 (Baseline(K)) respectively.

These results show that the correlation between the predictions of the different methods is relatively modest and that there is, as a consequence, significant scope for improving performance by combining the methods in a single predictive system. For example, it would be relatively easy to develop a high-recall system by naively combining AkanePPI(A), AkanePPI(B) and Baseline(K) (this would give recalls of 90% and 87% for HPRD50 and IEPA respectively), although it would also generate large numbers of false positives.

We undertook a manual analysis of these results and identified the following key points:

• *Easy interactions (joint true positives)*. As expected, the interactions correctly identified by all three systems consist of relatively simple sentences containing an interaction verb that is on the Baseline(K) list, for example the interactions between *A (beta) (1–40) *and *PIP2-PLC *in the following sentence from the IEPA corpus: "Moreover, A(beta) (1–40) significantly decreased the basal activity of the PIP2-PLC in SPM and the enzyme activity regulated through cholinergic receptors."

• *Illusory interactions (joint false positives)*. Of the 14 false positive interactions for both corpora, 6 are attributable to negation, i.e. where the sentence says that two genes/proteins do *not *interact. For example, the interactions between *A beta *and *PI-PLC*, and *A beta *and *PIP2-PLC *in the following sentence from the IEPA corpus: "Moreover, *A beta *25–35 had no effect on basal *PIP2-PLC *activity and cytosolic *PI-PLC *and *PIP2-PLC*."

The other joint false positive interactions may be attributable to sentence complexity. Hence, for example, the predicted interaction between *leptin *and *NPY *in the sentence: "Significantly increased *leptin *and galanin levels in postmenopausal obese women coupled with decreased *NPY *levels revealed some changes in the neuropeptides regulating eating behavior, which may be the reason for the onset of postmenopausal obesity."

• *Elusive interactions (joint false negatives)*. Manual analysis of a subset of the jointly missed interactions indicates that a large proportion are associated with sentences describing a specific set of processes that includes cross-linking, immunopercipitation with antibodies, cross talk and immunolocalisation. For example, all the tools missed the interaction between *CB1 *and *orexin 1 receptor *and/or *OX1R *in the sentence "In the present study, we observed evidence of cross-talk between the cannabinoid receptor *CB1 *and the *orexin 1 receptor *(*OX1R*) using a heterologous system."

• *The strengths and weaknesses of Baseline(K)*. The baseline algorithm often correctly retrieves interactions from complex sentences that the other methods failed to parse successfully, for example the interaction between *CLIP-170 *and *phospho-LIS1 *in the sentence "Overexpression of *CLIP-170 *results in a zinc finger-dependent localization of a *phospho-LIS1 *isoform and dynactin to MT bundles, raising the possibility that CLIP-170 and LIS1 regulate dynein/dynactin binding to MTs." On the other hand, Baseline(K) is prone to generate false positives whenever there are many entities in a sentence, as it predicts an interaction between every pair of entities that are separated by an interaction keyword.

### The effect of NER on GPI extraction

The effects of using the BANNER gene/protein tagger on the performance of AkanePPI(A) and AkanePPI(B) are shown in tables 5 and 6 respectively. These results show that using the BANNER tagger rather than the gold-standard entities leads to a significant drop in performance, with a fall of around 20 percentage points being commonplace. The negative impact of using BANNER is broadly correlated with the performance of AkanePPI on a given corpus; the better its performance using gold-standard entities, the greater the negative impact of using BANNER.

**Table 5:** The effect of using the BANNER entity tagger compared to gold-standard entities on the performance (precision, recall and F-score) of AkanePPI trained on AIMed.

	(A)	B	H	I	L
**Precision:**					

With gold-standard entities	57.0	29.2	61.5	60.2	69.6

With BANNER	34.3	23.8	26.8	13.0	39.8

Δ precision	22.7	5.4	34.7	47.2	29.8

**Recall:**					

With gold-standard entities	74.0	31.8	44.2	32.5	23.8

With BANNER	64.2	30.2	41.1	24.5	27.4

Δ recall	9.8	1.7	3.1	8.0	-3.7

**F-score:**					

With gold-standard entities	64.4	30.5	51.4	42.2	35.4

With BANNER	44.7	26.6	32.4	17.0	32.5

Δ F-score	19.7	3.8	19.0	25.2	3.0

Corpus abbreviations are as follows: A = AIMed; B = BioInfer; H = HPRD50; I = IEPA; L = LLL.

**Table 6:** The effect of using the BANNER entity tagger compared to gold-standard entities on the performance (precision, recall and F-score) of AkanePPI trained on BioInfer.

	A	(B)	H	I	L
**Precision:**					

With gold-standard entities	29.1	56.8	52.0	66.2	76.7

With BANNER	32.3	49.5	35.1	17.8	50.7

Δ precision	-3.2	7.3	16.9	48.4	26.0

**Recall:**					

With gold-standard entities	52.9	85.4	55.8	51.3	40.2

With BANNER	38.9	42.2	37.4	30.1	23.2

Δ recall	14.0	43.2	18.4	21.2	17.0

**F-score:**					

With gold-standard entities	37.5	68.2	53.8	57.8	52.8

With BANNER	35.3	45.5	36.2	22.4	31.8

Δ F-score	2.2	22.7	17.6	35.4	21.0

Corpus abbreviations are as follows: A = AIMed; B = BioInfer; H = HPRD50; I = IEPA; L = LLL.

Intuitively we expected the use of BANNER to have a greater impact on recall than precision on the grounds that AkanePPI would not be able to compensate for missed entities (false negatives), but would frequently be able to reject erroneous entities (false positives), as we expected the latter would often not be engaged in an apparent interaction. However, this was not the case in practice. Indeed, with AkanePPI(A) the drop in precision was always significantly greater than the drop in recall, with even a slight improvement in recall being experienced with the LLL corpus.

Of the 53 false positive gene and protein names identified by BANNER in the LLL corpus, 26 are involved in 46 relationships that AkanePPI(A) identifies as GPIs but which are not annotated as such in the corpus. Our manual analysis of these relationships shows that they fall into 3 main categories:

1. *Invalid interactions involving invalid names*. Only two of the 26 putative names tagged by BANNER are not the names of genes or proteins: "ShaA mutant" (a bacterium), and "AsiA form bacteriophage T4" (a virus). These two names participate in five of the 46 false positive interactions.

2. *Invalid interactions involving valid names*. In these cases BANNER tagged a valid gene or protein name that had not been annotated in the LLL corpus, but one or more interactions identified by AkanePPI involving this gene or protein are nevertheless invalid. This situation accounts for 29 of the 46 false positive interactions. Here is an example (with the names that are annotated in the LLL corpus italicized):

DNase I footprinting showed that *SpoIIID *binds strongly to two sites in the *cotC *promoter region, binds weakly to one site in the *cotX *promoter, and does not bind specifically to *cotB*.

In this case BANNER additionally tagged "DNase I" and AkanePPI identified an erroneous interaction between "DNAse I" and "CotB".

3. *More-or-less valid interactions involving valid names*. This situation accounts for 12 of the 46 false positive interactions. In these cases the LLL corpus has failed to annotate an essentially valid interaction because:

• There is an alternative, more specific name available. Take for example the phrase

The *sigma W *regulon includes a penicillin binding protein (PBP4*) and a co-transcribed amino acid racemase (*RacX*)...

Here BANNER tags "penicillin binding protein" instead of "PBP4 *" and an interaction between "sigma W regulon" and "penicillin binding protein" is annotated by AkanePPI.

• A gene or protein name is deemed to be too general by the LLL corpus. For example,

Our data demonstrate that the *CtsR *protein acts as a global repressor of the *clpC *operon, as well as other class III heat shock genes...

Here BANNER additionally tags "class III heat shock genes".

• The gene or protein is arguably involved in an uninteresting, though valid, interaction. For example:

In contrast, *sspJ *is transcribed in the forespore compartment by RNA polymerase with the forespore-specific *sigmaG *and appears to give a monocistronic transcript.

Here BANNER tags "RNA polymerase" AkanePPI identifies an interaction between "RNA polymerase" and "sspJ", an interaction that LLL presumably ignores because it is uninteresting.

The fact that only 12 of the 46 interactions that arise from false positive names are, in fact, valid suggests that over-prediction by gene/protein name taggers is potentially a serious problem. An analysis by Pyysalo and coworkers [1] appears to shed light on this effect. Their analysis suggests that the performance of GPI extraction methods is correlated with the I/EP of a given corpus, where I is the average number of interactions per sentence and EP is the average number of entity pairs per sentence [1]; roughly speaking, the smaller the value of I/EP, the more difficult the corresponding GPI extraction task. They go on to point out that, "As more proteins are annotated, we would not expect I to grow more than linearly, while EP grows quadratically". In this context, the potentially damaging effect of false positive predictions by a name tagger such as BANNER is clear (even where a given "false positive" name may be judged a valid gene or protein name) – the growth in the number of entity pairs makes the GPI extraction task more difficult.

It is worth noting that in the vast majority of these cases where the entities tagged by BANNER lead to AkanePPI(A) extracting a false positive interaction, the use of gold standard entities does not lead to the detection of the correct interaction, even though AkanePPI(A) is able to avoid tagging the incorrect interaction. In other words, entities tagged by BANNER are primarily responsible for increasing the numbers of false positives, and not for preventing the detection of true positives with AkanePPI(A). This is consistent with the full set of results for AkanePPI(A) on multiple corpora in table 5, which show a modest drop in recall with entities tagged by BANNER, but a large drop in precision.

One additional result that requires further explanation concerns the performance of AkanePPI(A) on the LLL corpus; AkanePPI(A) had higher recall with tagged entities than with gold standard ones (see table 5) – in spite of the fact that BANNER missed 27 genes/proteins (it correctly tagged 212 of the 239 genes/proteins in the LLL corpus). On manual inspection it was apparent that the improved recall is attributable to BANNER's tendency to tag longer versions of gene and protein names than appear in the gold standard annotations. For example, BANNER tags "cotX promoter" instead of "cotX" and, more dramatically, "IFN alpha tyrosine kinase Tyk-2" instead of "Tyk 2".

This is potentially significant because GPI prediction methods typically inherit a single token instead of the original, potentially multi-word name from the tagger; hence in cases such as these, AkanePPI is making predictions for sentences with a somewhat simpler – and sometimes much simpler – structure than that of the original text. In specific cases (such as the "cotX promoter" example given above), this enables both versions of AkanePPI to make correct predictions where they are unable to do so when the sentence is in its original, more complicated, form. However, the drop in recall for AkanePPI(B) is consistently high, so in practice this effect does not appear to be highly significant.

Finally, the effect of using the BANNER NER tagger on the performance of our simple baseline algorithm is shown in table 7. As with AkanePPI, the smallest drop in performance is on AIMed and BioInfer, for which the original performance was lowest. It is also worth noting that recall is on average no worse than that for AkanePPI, even though there is no scope for elongated name tagging to be beneficial with our benchmark algorithm.

**Table 7:** The effect of using the BANNER entity tagger compared to gold-standard entities on the performance (precision, recall and F-score) of our simple baseline algorithm, Baseline(K).

	A	B	H	I	(L)
**Precision:**					

With gold-standard entities	22.8	24	54	44.8	53.9

With BANNER	18.4	23.5	32	20	43.8

Δ precision	4.4	0.5	22	24.8	10.1

**Recall:**					

With gold-standard entities	51.5	52.2	66.9	56.4	72

With BANNER	42.1	33.5	49.7	27.5	51.2

Δ recall	9.4	18.7	17.2	28.9	20.8

**F-score:**					

With gold-standard entities	31.6	32.9	59.7	49.9	61.6

With BANNER	25.6	27.6	38.9	23.1	47.2

Δ F-score	6	5.3	20.8	26.8	14.4

Corpus abbreviations are as follows: A = AIMed; B = BioInfer; H = HPRD50; I = IEPA; L = LLL.

### Performance of the Whatizit protein interaction pipeline

To evaluate the Whatizit protein interaction pipeline, we adopted a slightly different approach for the reasons given above. We used un-annotated text as input and then scored the putative interactions generated by the pipeline against the gold standard entities and interactions in the evaluation corpora. In the case of sentences containing multiple occurrences of the same protein name, the pipeline was given full credit for identifying the correct name even though the precise context was not specified.

Results for the three Whatizit interaction detection methods – Ppi, Co3 and Co – are given in tables 8, 9 and 10 respectively. These results indicate that the Ppi method produces very low recall, ranging from 0.3% on IEPA to 4.4% on AIMed (table 8). Although the Co3 approach is very similar to our Baseline(K) algorithm, it achieves significantly lower performance. This is attributable to the much lower rate of entity detection with Whatizit, a consequence of its requirement that only names it is able to map to UniProt identifiers are tagged.

**Table 8:** Performance of the Whatizit protein interaction pipeline, Ppi method.

	A	B	H	I	L
Precision	73.8	58.6	83.3	25.0	1.0

Recall	4.4	1.4	3.2	0.3	1.3

F-score	8.3	2.7	6.1	0.6	2.5

Corpus abbreviations are as follows: A = AIMed; B = BioInfer; H = HPRD50; I = IEPA; L = LLL.

**Table 9:** Performance of the Whatizit protein interaction pipeline, Co3 method.

	A	B	H	I	L
Precision	29.3	31.3	24.5	12.4	31.8

Recall	14.5	10.7	15.9	4.6	8.8

F-score	19.4	15.9	19.3	6.7	13.8

Corpus abbreviations are as follows: A = AIMed; B = BioInfer; H = HPRD50; I = IEPA; L = LLL.

**Table 10:** Performance of the Whatizit protein interaction pipeline, Co method.

	A	B	H	I	L
Precision	21.8	16.2	21.4	10.2	25.3

Recall	52.1	49.8	49.7	42.1	31.4

F-score	30.7	24.4	29.9	16.4	28.0

Corpus abbreviations are as follows: A = AIMed; B = BioInfer; H = HPRD50; I = IEPA; L = LLL.

Of the three approaches, the simple name co-occurrence (Co) approach gives the best results. In the case of Co, the low rate of Whatizit entity detection actually proves beneficial, as it reduces the number of false positive interactions that would otherwise be detected using this naïve approach.

## Conclusion

In this paper we have uncovered some sobering facts about the current state of automated GPI extraction. Firstly, in spite of all the research that has been undertaken to develop relatively sophisticated GPI extraction methods using grammatical parsers, we have concluded (after considerable effort and having consulted widely) that, with the exception of the Whatizit protein interaction pipeline accessed via its web interface, none of the tools are easy to install and evaluate. Although AkanePPI performs significantly better than OpenDMAP, what is most startling is that both tools generally perform worse (in terms of F-score on a range of GPI corpora) than a simple keyword-based approach using regular expressions. (We exclude Whatizit from this judgement as its stringent requirement that only names mapped to UniProt identifiers are tagged makes a fair comparison impossible.) Better-performing tools have already been developed (such as the rule-based RelEx), but are not available to the vast majority of potential users.

Secondly, we have quantified the effect of using a state-of-the-art NER tagger on the performance of three GPI extraction methods when evaluated on standard corpora. A drop in F-score of over 15 percentage points occurred in 10 out of 15 cases. Moreover, when the performance of the GPI extraction method is at its best, the typical drop is even greater – a point that is worth bearing in mind when reading reports of high-performing GPI methods. For example, if we take the two best F-scores for each of the extraction methods, the average drop in F-score with BANNER is more than 20 percentage points.

These results emphasize two points – the urgent need to make the best tools publicly available, and the need to carry out realistic evaluations of tools using name taggers and multiple corpora.

Finally, this paper has identified three areas that may prove fertile for additional research. Firstly, given the significant impact of gene/protein tagging on GPI extraction performance, we think it worth investigating whether other gene/protein name taggers produce less significant drops in performance than BANNER. Although we show that BANNER is the best available tagger in terms of general performance on the gene/protein named entity recognition task, this does not necessarily mean that it is the best tagger for GPI extraction.

Secondly, our joint error analysis of three tools shows that very high levels of recall (around 90%) would be achievable using a system that combined all three of them, suggesting that hybrid systems may prove highly effective. (Indeed, this is what Whatizit already does in the context of mapping names to UniProt identifiers.)

Thirdly, our baseline approach using Perl regular expressions was deliberately simple. Given that it performed surprisingly well compared to more sophisticated tools and that it correctly identifies many interactions not found by other methods, we believe that it would be worthwhile devising and evaluating more complicated approaches that exploit simple regular expressions.

## Authors' contributions

RK designed and wrote the scripts used in the experiment, analyzed the results and drafted the manuscript. ABC devised the keyword-based benchmark algorithm and set up and executed OpenDMAP. AJS participated fully in the experimental design and data analysis, and contributed to the writing of the manuscript. All three authors co-edited, read and approved the final manuscript.

## Supplementary Material

Additional file 1 **GPI Keywords**. A text file containing the list of interaction keywords used by the Baseline(K) method. Click here for file

Additional file 2 **Baseline K Perl Script**. A Perl script that implements the Baseline(K) method. To be used in conjunction with the keyverbs.txt file. Zipped PL file Click here for file
